# Association of Habitual Physical Activity Measured by an Accelerometer with High-Density Lipoprotein Cholesterol Levels in Maintenance Hemodialysis Patients

**DOI:** 10.1155/2013/780783

**Published:** 2013-12-21

**Authors:** Ryota Matsuzawa, Atsuhiko Matsunaga, Toshiki Kutsuna, Akira Ishii, Yoshifumi Abe, Kei Yoneki, Manae Harada, Mio Ishibashi, Yasuo Takeuchi, Atsushi Yoshida, Naonobu Takahira

**Affiliations:** ^1^Department of Rehabilitation Sciences, Graduate School of Medical Sciences, Kitasato University, 1-15-1 Kitasato, Sagamihara, Kanagawa 252-0373, Japan; ^2^Hemodialysis Center, Sagami Junkanki Clinic, Sagamihara, Japan; ^3^Rehabilitation Center, Kitasato University East Hospital, Sagamihara, Japan; ^4^Department of Cardioangiology, Graduate School of Medical Sciences, Kitasato University, 1-15-1 Kitasato, Sagamihara, Kanagawa 252-0373, Japan; ^5^Division of Nephrology, Department of Internal Medicine, Kitasato University School of Medicine, Sagamihara, Japan

## Abstract

After confirming the relationship between high-density lipoprotein cholesterol (HDL-C) levels and mortality in hemodialysis patients for study 1, we investigated the effect of physical activity on their HDL-C levels for study 2. In study 1, 266 hemodialysis patients were monitored prospectively for five years, and Cox proportional hazard regression confirmed the contribution of HDL-C to mortality. In study 2, 116 patients were recruited after excluding those with severe comorbidities or requiring assistance from another person to walk. Baseline characteristics, such as demographic factors, physical constitution, primary kidney disease, comorbid conditions, smoking habits, drug use, and laboratory parameters, were collected from patient hospital records. An accelerometer measured physical activity as the number of steps per day over five consecutive days, and multiple regression evaluated the association between physical activity and HDL-C levels. Seventy-seven patients died during the follow-up period. In study 1, we confirmed that HDL-C level was a significant predictor of mortality (*P* = 0.03). After adjusting for patient characteristics in study 2, physical activity was independently associated with HDL-C levels (adjusted *R* ^2^ = 0.255; *P* = 0.005). In conclusion, physical inactivity was strongly associated with decreased HDL-C levels in hemodialysis patients.

## 1. Introduction

The mortality rate of hemodialysis patients remains high despite continued improvements in dialysis technology. Cardiac disease is the leading cause of death among maintenance hemodialysis patients, accounting for approximately 38.1% of reported deaths in the United States [1] and 35% of deaths in Japan [2]. The relative risk of death from cardiovascular disease is reportedly 10 to 20 times greater in these patients than the general population [3]. Individuals with only low kidney function tend to have a high risk of developing generalized atherosclerosis and cardiovascular disease [4], while hemodialysis patients carry several additional risk factors. Although the underlying mechanisms for increased cardiovascular risk in dialysis patients are not completely understood, lipid disorder is one of the more well-known risk factors.

Shoji et al. examined the relationship between lipid parameters, cardiovascular events, and all-cause mortality in patients from a large cohort run by the Japanese Society for Dialysis Therapy [5]. After adjusting for clinical variables, high-density lipoprotein cholesterol (HDL-C) levels were significantly and inversely associated with risks of incident myocardial infarction, cerebral infarction, and all-cause mortality in hemodialysis patients. Keeping HDL-C levels within a normal range may thus be important for disease management.

The best approach to modify HDL-C levels in dialysis patients is uncertain, although some studies found that physical activity increased HDL-C levels in general populations [6–9]. In a previous meta-analysis, Kodama et al. examined the relationship between physical activity, represented as walking or jogging, and HDL-C levels in the general population. Their findings showed that exercise resulted in a 2.53 mg/dL increase in HDL-C levels [10]. Physical activity could be similarly effective on HDL-C levels in dialysis populations.

The present study includes both confirmation and evaluation aspects. After first confirming the relationship between HDL-C levels and all-cause mortality in hemodialysis patients for study 1, we then investigated the effect of habitual physical activity on HDL-C levels in a cohort of clinically stable and adequately dialyzed patients for study 2.

## 2. Materials and Methods

### 2.1. Study Population

In study 1, 266 patients at the Hemodialysis Center at Sagami Junkanki Clinic in July 2008 were included in a prospective study and monitored for five years. In study 2, 116 patients were recruited from the same pool for a cross-sectional study with the following exclusion criteria: hospitalization within three months prior to the study; recent myocardial infarction or angina pectoris; uncontrolled cardiac arrhythmias, hemodynamic instabilities, uncontrolled hypertension, or renal osteodystrophy with severe arthralgia; or assistance by another person to walk.

The studies were approved by the Kitasato University Allied Health Sciences Research Ethics Committee. Physicians obtained informed consent from all patients.

### 2.2. Demographic and Clinical Factors

In study 1, demographic factors (age, sex, and time on hemodialysis) and lipid parameters (serum HDL-C, serum low-density lipoprotein cholesterol (LDL-C), and serum triglycerides (TG)) were collected at the time of patient entry.

In study 2, demographic factors (age, sex, and time on hemodialysis), physical constitution (body mass index), primary kidney disease, comorbid condition (cardiac disease, diabetes mellitus), smoking habits (Brinkman index), cardiovascular medications (antilipemics, angiotensin converting enzyme inhibitor, angiotensin receptor blocker, calcium channel blocker, and beta blocker), laboratory parameters (serum albumin, serum creatinine, serum hemoglobin, hematocrit, serum phosphorus, serum calcium, intact parathyroid hormone (intact PTH), serum HDL-C, serum LDL-C, and serum TG), and habitual physical activity were collected from patient hospital charts. To quantify comorbid illnesses, a comorbidity index was developed for dialysis patients. The score was calculated with a method previously used to analyze survival in hemodialysis patients [11].

### 2.3. Habitual Physical Activity

An accelerometer pedometer (Lifecorder; Suzuken Co., Ltd., Nagoya, Japan) was used to measure the habitual physical activity of patients in study 2. The device obtains objective information on physical activity patterns because it continuously measures the intensity, duration, and frequency of activities. The accuracy and reliability of this instrument have been reported in previous studies [12, 13]. Because the monitor does not capture activities such as use of a stationary cycle, those activities were confirmed via interview at each patient's followup.

The accelerometer for this study was worn around the waist, where it translated acceleration of the body as motion that was recorded as number of steps taken. Patients were instructed to wear the device continuously during waking hours for seven days and avoid getting it wet. Patients were also asked to maintain their typical weekly schedules. To ensure that measurement periods were typical of their weekly activity patterns, data were excluded when patients traveled or manifested an acute illness.

Prior to analysis, accelerometer data were inspected to ensure that there were no obvious errors, such as failure to acquire data or wear the device. Measurements from a consecutive 5-day period were analyzed.

### 2.4. Statistical Analysis

Data are presented as medians (25th, 75th percentile) or number (%) and tested by the Mann-Whitney *U* test or chi-square test. In study 1, patients were categorized into four groups by quartile, and survival rate differences between groups were tested using the log-rank test. Multivariate analysis was performed with the Cox proportional hazards regression model to confirm the contribution of HDL-C levels to survival after adjusting for confounders. In study 2, physical activity was evaluated with an accelerometer as the number of steps per day for a consecutive 5-day period. Number of steps was used as the index of habitual physical activity because it was generalizable to daily practice. Patients were categorized into two groups by a physical activity median value, and the difference between groups was tested using the Mann-Whitney *U* test or chi-square test. Multiple linear regression analysis was used to evaluate the relationship between physical activity and HDL-C levels. *P* < 0.05 was considered statistically significant. Analyses were performed using SPSS version 12.0 software (IBM Corporation, Armonk, NY, USA).

## 3. Results

### 3.1. Study 1: Lipid Parameters and Survival

Baseline demographic and clinical factors for the patients in study 1 are summarized in Table 1. The 266 patients (163 men, 103 women) ranged in age from 28 to 93 years (median, 65 years). Time on hemodialysis ranged from 3.0 to 404.0 months (median, 80.0 months). The median values of HDL-C, LDL-C, and TG were 41.0 mg/dL, 83.5 mg/dL, and 106.5 mg/dL, respectively.

**Figure 1:**
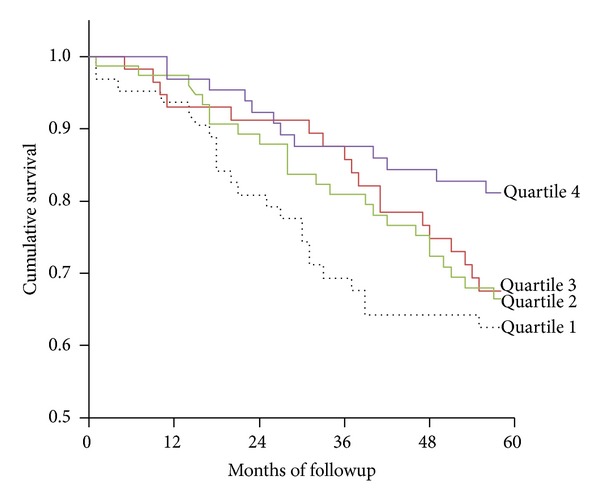
Kaplan-Meier analysis of survival for 266 hemodialysis patients. The survival rate of patients in the highest quartile (quartile 4) of HDL-C was significantly higher than that of patients in the lowest quartile (quartile 1) (*P* = 0.02 by log-rank test).

Patients were followed for five years, with 77 deceased at the end of the follow-up period. The 5-year cumulative survival rates in quartiles 1, 2, 3, and 4 were more than half at 64.1%, 69.0%, 68.8%, and 82.1%, respectively. A total of 25% of the patients in quartiles 1, 2, and 3 died after 31, 48, and 42 months, respectively. This statistic was not applicable for patients in quartile 4, indicating greater survival in patients with the highest HDL-C levels (Figure 1).

Using the Cox proportional hazards model, the crude hazard ratio of HDL-C increased per mg/dL was 0.97 (95% confidence interval (CI), 0.96–0.99; *P* = 0.005), which indicated that maintenance of high HDL-C levels was associated with a reduction in all-cause mortality. After adjusting for age, sex, time on hemodialysis, LDL-C levels, and TG levels, the hazard ratio was 0.98 (95% CI, 0.95–0.99; *P* = 0.03) (Table 2).

### 3.2. Study 2: Effect of Physical Activity on HDL-C Levels

Table 3 shows baseline characteristics and results for the 116 patients in study 2, with physical activity < median value and ≥ median value. The most common underlying kidney diseases were diabetic nephropathy (35.3%) and glomerulonephritis (34.5%), and the median comorbidity score was 6.0 (3.3, 8.0). The median number of steps taken was 3208 (1828, 4481). Only one patient participated in the activity of cycling or swimming. Patients in the group of ≥ median value were significantly younger than the group of < median value (*P* = 0.04). HDL-C levels in patients with physical activity ≥ median value were significantly higher than the < median value group (*P* = 0.001), and intact PTH and TG levels were significantly lower (*P* = 0.04). There were no significant differences in other characteristics between groups.

Parameters such as age, sex, body mass index, time on hemodialysis, presence of diabetes mellitus, albumin, creatinine, intact PTH, LDL-C, TG, treatment of lipid-modifying medication, Brinkman index, and physical activity were used as explanatory variables in the multiple linear regression analysis for HDL-C levels. As a result, only TG, age, body mass index, and physical activity were selected stepwise in multivariate analysis as significant (*R* ^2^ = 0.40) (Table 4). After adjustments, greater physical activity was significantly associated with increased HDL-C levels.

## 4. Discussion

In the present study, we reported the significant effect of physical activity on HDL-C levels, which was confirmed to be a risk factor for mortality in hemodialysis patients, independent of age, sex, body mass index, time on hemodialysis, presence of diabetes mellitus, albumin, creatinine, intact PTH, LDL-C, TG, lipid-modifying medications, and the Brinkman index. This report is the first to show a correlation between physical activity measured by accelerometer and HDL-C levels in hemodialysis patients. Based on our findings, greater amounts of physical activity are associated with a reduced risk for death in dialysis populations and could even improve prognosis.

Our study reconfirmed that low HDL-C blood levels were a strong risk factor for death in hemodialysis patients, as previously reported [5]. However, Briel et al. systematically reviewed the association of HDL-C change induced by medication with outcomes in nondialysis patients and reported that the risks of coronary heart disease events, coronary heart disease deaths, and total deaths were not reduced [14]. There are two possible reasons for this observation. First, the mean baseline value of HDL-C in patients was 47.3 mg/dL, meeting the concentration recommended as a lipid management goal by the 2007 Japan Atherosclerosis Society Guideline for Diagnosis and Prevention of Atherosclerotic Cardiovascular Disease for Japanese (≥40 mg/dL) [15]. The median value of HDL-C in our study population was 41.0 mg/dL, which was below the recommended levels for approximately half of the patients. Second, the weighted mean change of HDL-C in the report was only 1.7 mg/dL, which indicates that cardiovascular medications may not be very effective in significantly increasing HDL-C levels. Other published data, however, indicate that physical activity can modify HDL-C levels.

Goldberg et al. examined the metabolic effects of exercise training in hemodialysis patients and reported an increase in HDL-C levels [16, 17]. The high intensity habitual exercise of that study does not appear realistic, however, because of the adverse symptoms related to hemodialysis, low physical function or general poor adherence to regular exercise programs [18–22]. Yano et al. instead studied the relationship between HDL-C levels and physical activity as measured by steps per day, which hemodialysis patients could participate in reasonably and comfortably [23]. Unfortunately, the small sample size of 35 patients and lack of confounding factors gave the study less reliability. We therefore evaluated habitual physical activity using an accelerometer and reexamined the effect of physical activity on HDL-C levels in 116 hemodialysis patients. The median number of steps our patients walked was approximately 3000 steps/day, a number markedly lower than for healthy controls [24, 25] and more similar to patients with chronic disease [26]. Our results showed that physical activity was significantly and positively associated with HDL-C levels after adjusting for the effects of confounders. We had previously showed the correlation between physical inactivity and higher mortality in a cohort of patients on maintenance hemodialysis [27]. Physical activity was evaluated using an accelerometer and this method was the same method as in the present study. In the study, the underlying mechanisms remained to be elucidated. However, the present study results may determine a part of the potential mechanisms. It might be that physical inactivity in patients on maintenance hemodialysis decreased their HDL-C levels and potentially worsened their prognosis.

Two possible reasons can explain the results of the present study. First, physical activity may increase HDL-C levels in hemodialysis patients partly by increasing insulin sensitivity. Insulin resistance tends to gain TG levels and reduce lipoprotein lipase activity, which boost HDL-C levels. In a previous study, exercise interventions improved the insulin sensitivity of patients afflicted with lifestyle-related diseases such as type 2 diabetes and hypertension [28]. Goldberg et al. also examined the association between physical activity and insulin sensitivity, reporting that improvements in insulin sensitivity could affect lipid abnormalities observed in dialysis patients [17]. Second, patients with adequate physical activity often have good nutrition. Malnutrition is a predictor of mortality in dialysis patients [29] and strongly associated with decreased HDL-C levels. Patients in this study with decreased physical activity may suffer from malnutrition as well as low HDL-C.

There are some limitations to this study. First, residual confounders remain possible because the study was observational. To our knowledge, this is the first study to identify physical activity as a strong predictor of HDL-C levels by using multivariate analysis adjusted for confounding factors. However, further randomized, controlled studies are still needed. Second, we excluded patients who needed assistance with walking. As a result, the severity of comorbidities in participants seemed mild. This should be considered when generalizing our results to more severely limited patients. Third, alcohol intake can influence HDL-C levels but was not included in our study. This was because there were no patients who consumed excessive alcohol in our study, as most hemodialysis patients are conscious about becoming overhydrated. Finally, although we reported lower HDL-C levels in hemodialysis patients with lower physical activity, the underlying mechanisms have yet to be elucidated.

## 5. Conclusion

Physical activity is strongly associated with increased HDL-C levels in hemodialysis patients. Although we believe it is important for these patients to engage in physical activities, further studies are needed to determine the possible mechanisms.

## Figures and Tables

**Table 1:** Baseline patient characteristics.

	*N* = 266
Age, years	65 (57, 72)
Gender, women	103 (38.7%)
Time on hemodialysis, months	80.0 (26.0, 172.3)
Laboratory parameters	
HDL-C, mg/dL	41.0 (34.0, 53.0)
LDL-C, mg/dL	83.5 (65.8, 99.0)
Triglycerides, mg/dL	106.5 (83.0, 151.3)

Values are expressed as median (25th, 75th percentiles) or number (percentage of total).

HDL-C: high-density lipoprotein cholesterol; LDL-C: low-density lipoprotein cholesterol.

**Table 2:** Univariate and multivariate analysis for associations with all-cause mortality.

Factors	Units of increase	Univariate analysis		Multivariate analysis	
		HR (95% CI)	*P *	HR (95% CI)	*P *
Age, years	1 year	1.07 (1.05–1.09)	<0.001	1.07 (1.04–1.10)	<0.001
Women versus men	—	0.57 (0.35–0.93)	0.03	0.64 (0.39–1.07)	0.09
Time on hemodialysis, months	1 month	0.99 (0.99–1.00)	0.03	1.00 (0.99–1.00)	0.9
HDL-C, mg/dL	1 mg/dL	0.97 (0.96–0.99)	0.005	0.98 (0.95–0.99)	0.03
LDL-C, mg/dL	1 mg/dL	1.00 (0.99–1.01)	0.9	0.99 (0.99–1.01)	0.4
Triglycerides, mg/dL	1 mg/dL	0.99 (0.99–1.00)	0.2	0.99 (0.99–1.00)	0.3

Analyses were performed using a Cox proportional hazards regression model.

HR: hazard ratio; CI: confidence interval; HDL-C: high-density lipoprotein cholesterol; LDL-C: low-density lipoprotein cholesterol.

**Table 3:** Baseline characteristics in patients with < median physical activity and ≥ median physical activity.

	All patients (*n* = 116)	Physical activity (steps/day)		*P *
		< Median value (*n* = 58)	≥ Median value (*n* = 58)	
Age, years	68 (62, 74)	70 (64, 75)	66 (61, 71)	0.04
Gender, women	58 (50.0%)	26 (44.8%)	32 (55.2%)	0.3
Body mass index, kg/m^2^	20.9 (19.1, 23.3)	20.3 (18.9, 24.2)	21.1 (19.5, 22.8)	0.7
Time on hemodialysis, months	66.5 (34.3, 150.8)	73.0 (34.3,147.0)	65.5 (33.5, 164.8)	0.9
Primary kidney disease, %				0.6
Diabetic nephropathy	41 (35.3%)	20 (34.5%)	21 (36.2%)	
Glomerulonephritis	40 (34.5%)	19 (32.8%)	21 (36.2%)	
Hypertension	6 (5.2%)	2 (3.4%)	4 (6.9%)	
Polycystic renal disease	5 (4.3%)	4 (6.9%)	1 (1.7%)	
Other nephropathies	11 (9.5%)	7 (12.1%)	4 (6.9%)	
Unknown	13 (11.2%)	6 (10.3%)	7 (12.1%)	
Comorbid condition, %				
Presence of cardiac disease	31 (26.7%)	16 (27.6%)	15 (25.9%)	0.8
Presence of diabetes mellitus	57 (49.1%)	32 (55.2%)	25 (43.1%)	0.2
Comorbidity score	6.0 (3.3, 8.0)	6.0 (4.0, 8.0)	6.0 (3.0, 7.0)	0.5
Laboratory parameters				
Albumin, g/dL	3.8 (3.6, 3.9)	3.8 (3.6, 3.9)	3.8 (3.6, 4.0)	0.8
Creatinine, mg/dL	10.2 (8.9, 11.4)	10.3 (8.7, 11.2)	10.2 (9.0, 11.6)	0.7
Hemoglobin, g/dL	11.1 (10.7, 11.9)	11.1 (10.7, 11.7)	11.2 (10.7, 11.9)	0.7
Hematocrit, %	33.2 (31.9, 34.9)	33.2 (31.5, 34.6)	33.3 (32.0, 35.4)	0.3
Phosphorus, mg/dL	5.0 (4.2, 5.7)	5.1 (4.3, 5.7)	5.0 (4.2, 5.6)	0.4
Calcium, mg/dL	8.7 (8.4, 9.2)	8.8 (8.5, 9.1)	8.6 (8.4, 9.2)	0.2
Intact PTH, pg/mL	104.0 (57.5, 184.0)	127.0 (69.5, 208.5)	85.5 (40.0, 165.8)	0.04
HDL-C, mg/dL	41.0 (32.0, 52.5)	37.0 (30.0, 47.0)	47.0 (38.0, 57.0)	0.001
LDL-C, mg/dL	88.0 (70.0, 108.5)	84.0 (65.5, 103.5)	91.5 (71.5, 115.0)	0.1
Triglycerides, mg/dL	119.0 (82.0, 159.5)	136.0 (92.5, 179.5)	112.5 (69.0, 143.3)	0.04
Medications, %				
ACEI	6 (5.2%)	3 (5.2%)	3 (5.2%)	1.0
ARB	58 (50.0%)	34 (58.6%)	24 (41.4%)	0.06
CCB	52 (44.8)	26 (44.8%)	26 (44.8%)	1.0
Beta-blocker	32 (27.6%)	20 (34.5%)	12 (20.7%)	0.1
Lipid-modifier	12 (10.3%)	6 (10.3%)	6 (10.3%)	1.0
Smoking, %				0.7
Current	12 (10.3%)	7 (12.1%)	5 (8.6%)	
Past	56 (48.3%)	27 (46.6%)	29 (50.0%)	
Brinkman index	60.0 (0.0, 600.0)	225.0 (0.0, 787.5)	40.0 (0.0, 600)	0.5
Physical activity				
Number of steps	3208 (1828, 4481)	1833 (1185, 2382)	4478 (3726, 6242)	<0.001

Values are expressed as median (25th, 75th percentiles) or number (percentage) of patients.

Intact PTH: intact parathyroid hormone; HDL-C: high-density lipoprotein cholesterol; LDL-C: low-density lipoprotein cholesterol; ACEI: angiotensin converting enzyme inhibitor; ARB: angiotensin receptor blocker; CCB: calcium channel blocker.

**Table 4:** Prediction models for level of high-density lipoprotein cholesterol.

Model	Standardized coefficients (*β*)	*P* value	*R* ^2^	*R* ^2^ change
High-density lipoprotein cholesterol			0.40	
Triglycerides, mg/dL	−0.39	<0.001		0.24
Age, years	−0.28	<0.001		0.08
Body mass index, kg/m^2^	−0.26	0.003		0.05
Physical activity, steps/day	0.18	0.02		0.03

Analyses were performed using multiple linear regression analysis.
